# Challenges and Pitfalls in the Engineering of Human Interleukin 22 (hIL-22) Secreting *Lactobacillus reuteri*

**DOI:** 10.3389/fbioe.2020.00543

**Published:** 2020-06-05

**Authors:** Laura Ortiz-Velez, Annie Goodwin, Laura Schaefer, Robert A. Britton

**Affiliations:** ^1^Department of Molecular Virology and Microbiology, Baylor College of Medicine, Houston, TX, United States; ^2^Section of Pediatric Gastroenterology, Department of Pediatrics, Baylor College of Medicine, Texas Children's Hospital, Houston, TX, United States; ^3^Alkek Center for Metagenomics and Microbiome Research, Baylor College of Medicine, Houston, TX, United States

**Keywords:** lactobacilus, therapeutic delivery, IL-22, probiotic, secretion

## Abstract

Engineered microbes for the delivery of intestinally directed therapeutics is a promising avenue for the treatment of various intestinal diseases including inflammatory bowel disease (IBD) and intestinal graft vs. host disease (GVHD). This modality of treatment would allow for the targeted delivery of therapeutics to the site of inflammation or disease while minimizing the systemic side effects that often accompany treatment of these pathologies. Here, we show the challenges encountered and overcome in successfully engineering *Lactobacillus reuteri* to secrete high levels of biologically active human interleukin 22 (hIL-22). Initial hIL-22 constructs secreted high levels of hIL-22, however we found the majority of hIL-22 was cleaved and not biologically active. Several strategies were explored to improve the production of intact hIL-22, with the optimization of the signal sequence for peptide secretion having the most impact of production of intact hIL-22. This resulted in *L. reuteri* secreting high concentrations (up to 700 ng/mL) of hIL-22. Bioactivity of hIL-22 was confirmed by the secretion of interleukin 10 (IL-10) from the colon cancer derived epithelial cell line Colo205 and the secretion of Regenerating islet-derived protein 3 alpha (Reg3α) from human jejunal enteroids. The secretion of bioactive hIL-22 imposed a significant cost for *L. reuteri* as bacterial growth was significantly impaired upon induction. Future challenges and optimization strategies for the delivery of hIL-22 to the human intestinal tract are discussed.

## Introduction

Engineered commensal bacteria are an attractive alternative for providing the delivery of *in situ* biologics to the gastrointestinal tract (GIT) as they allow for targeted delivery of therapeutics directly to the area of inflammation or disease while minimizing systemic absorption and side effects. This method of drug delivery is especially pertinent to the treatment of diseases of the GIT including inflammatory bowel disease (IBD) and intestinal graft vs. host disease (GVHD), where current therapies involve use of systemic immunosuppressants that pose significant risks to the patient. Additionally, many of these microbes are adapted for survival in the harsh environment of the human GIT where they are able to sense and respond to various environmental signals. Two bacteria that have been extensively explored as a chassis for building therapeutic delivery systems include *Escherichia coli* Nissle and *Lactococcus lactis*. Several studies have demonstrated the ability of these strains to achieve beneficial effects by delivering therapeutic proteins. *E. coli* Nissle has previously been engineered to deliver the angiogenesis inhibitor Tumstatin (Tum-5) to restrain murine melanoma; recombinant strains of *L. lactis* have been engineered to deliver interleukin 10 (IL-10) and Elafin to ameliorate colitis in mice (Steidler et al., 2000; Bermudez-Humaran et al., 2015; He et al., 2017). These microbial delivery platforms, however, possess limitations. *E. coli* Nissle is a strain that can potentially cause adverse effects and disease when the host microbiome or immune system are compromised as shown in mouse studies wherein germ free *RAG1*^−/−^ mice had increased bacterial translocation and overall mortality when orally challenged with *E. coli* Nissle (Gronbach et al., 2010) Additionally, certain strains of *E. coli* harbor a genomic island called pks which encodes for the genotoxin colibactin, which has previously been shown to induce DNA damage *in vivo* and potentially play a role in sporadic colorectal cancer tumorigenesis (Cuevas-Ramos et al., 2010). In contrast, *L. lactis* does not survive passage through the GIT (only 10–30% of ingested bacteria survive in the duodenum) and its optimal growth temperature is different from the human body's temperature; which may limit its potential as a platform for the delivery of therapeutic molecules (Drouault et al., 1999; Kimoto et al., 2003).

Lactic acid bacteria (LAB) are a group of gram-positive bacteria characterized by their ability to produce lactic acid as a primary or secondary end-product of fermentation (Berlec et al., 2012; Wyszynska et al., 2015). Several members of this group have been used for decades as cell factories in the food production industry, which has allowed them to acquire the Generally Regarded As Safe (GRAS) status (given by the Federal Drug Administration) (WHO, 2001). *Lactobacillus reuteri* ATCC PTA 6475 is a human derived LAB that has an optimal growth temperature similar to that of the human body. It also grows in the presence of bile as well as survives in extremely acidic conditions (Wall et al., 2007; Whitehead et al., 2008). Interestingly, this bacterium has immunomodulatory properties that have shown benefits in several mouse models of disease including enterohemorrhagic *E. coli* infection, osteoporosis, obesity, and autism spectrum disorder (Eaton et al., 2011; Poutahidis et al., 2013; Buffington et al., 2016). Additionally, previous work by our group and others have developed genetic tools that facilitate the manipulation of *L. reuteri* 6475 genome, a crucial step to allow for the successful engineering and optimization of this strain (van Pijkeren and Britton, 2012; Van Pijkeren et al., 2012; Zhang et al., 2018). Taken together, these characteristics make *L. reuteri* 6475 an attractive choice for the delivery of therapeutic molecules to the GIT.

The therapeutic molecule we focused on is the cytokine interleukin-22 (IL-22) due to its unique roles in antimicrobial defense at gastrointestinal mucosal surfaces and wound healing. This cytokine is considered a master regulator of the intestinal barrier since it controls a wide range of effector molecules that preserve intestinal homeostasis, including antimicrobial peptides, mucus associated proteins, and other cytokines (Nagalakshmi et al., 2004; Dudakov et al., 2015; Lindemans et al., 2015; Mulcahy et al., 2016). This molecule also participates in wound healing after injury to the skin and intestine, promoting effects such as the proliferation and expansion of the intestinal stem cell compartment to accelerate epithelial regeneration (Lindemans et al., 2015). Additionally, the delivery of IL-22 has shown beneficial effects in the pathology of several animal models of disease including vancomycin resistant enterococcus infections, liver damage, diabetic wounds and inflammatory bowel disease, thus demonstrating its therapeutic potential (Zenewicz et al., 2008; Avitabile et al., 2015; Abt et al., 2016).

Previously *L. lactis* and *L. plantarum* have been engineered for the secretion of mouse IL-22 (mIL-22) and human (hIL-22), respectively. Recently, *L. reuteri* was engineered to successfully deliver murine IL-22 to the GIT; however, this delivery was achieved through the release of intracellular murine IL-22 after spontaneous cell lysis within the intestinal lumen (Hendrikx et al., 2019). Our aim was to engineer *L. reuteri* 6475 to produce and secrete intact and biologically active human IL-22 not only as a proof-of-concept that *L. reuteri* 6475 can be used as a platform for the delivery of therapeutic molecules, but also to investigate the therapeutic role of hIL-22 in an *ex vivo* model of human intestinal epithelium (human intestinal enteroids).

## Methods and Materials

### Bacterial Strains, Media

All bacterial strains used in this study are listed in Table 1 and Supplementary Table 1. Lactobacillus species were routinely cultured statically under anaerobic conditions at 37°C in de Man Rogosa and Sharpe (MRS) broth (Difco, BD Biosciences Cat # DF0882-17-0) or MRS agar plates (containing 1.5% Difco Bacto agar). Bacterial subcultures were done in buffered MRS (bMRS) which was prepared with the addition of 0.1M monobasic potassium phosphate (Fisher Cat # BP362-500) and 0.1M dibasic potassium phosphate (Fisher Cat # P288-500) to MRS with pH adjustment to 7.0. For cell culture assays, Lactobacillus species were grown in *L. reuteri* Cell Culture (LCC) media containing a mixture of 20% bMRS and 80% Roswell Park Memorial Institute medium (RPMI, ATCC 30-2001, containing 10% FBS). *Lactococcus lactis* was grown statically at 30°C in M17-broth (Difco, BD BioSciences), and supplemented with glucose to a final concentration of 0.5% (w/v). *Escherichia coli* 1000, the cloning host, were grown in Luria-Bertani (LB) at 37°C with vigorous shaking at 250 rpm. Antibiotics were added to the media, when required, at a concentration of 8 μg/ml, 5 μg/ml, and 300 μg/ml of erythromycin for *Lactobacilli* spp., *L. lactis*, and *E. coli*, respectively (Sorvig et al., 2005; Lizier et al., 2010).

**Table 1:** Primary strains and plasmids utilized.

**Strain**	**Description**	**Source**
*Lactobacillus reuteri* 6475	*L. reuteri* ATCC PTA 6475, Human breast milk isolate	Biogaia
PRB577	*L. reuteri* harboring pSIP411	This work
PRB484	*L. reuteri* harboring pLOV33	This work
PRB782	*L. reuteri* harboring pLS103	This work
PRB873	*L. lactis* harboring pLS107	This work
**Plasmid**	**Description**	**Source**
pSIP411	pSH71 replicon, gusA gene (glucuronidase), Sakacin inducible system; antibiotic resistance marker: erythromycin	Sorvig et al., 2005
pLOV33	pSIP411 with hIL-22 with Signal Peptide (SP000) for inducible expression of hIL-22	This work
pLS103	pLOV33, SP000 was replaced by SP004, inducible expression of hIL-22	This work
pLOV02	pSIP411 for inducible expression of Elafin (SP000_Elafin)	This work
pNZ8048	pSH71 replicon with the nisin inducible expression system; antibiotic resistance marker chloramphenicol	
PLS107	pNZ8048 expressing the usp45 signal peptide (from *L. lactis*) fused to the hIL-22 sequence, codon optimized for expression in *L. lactis*	

### Construction of the Inducible and Constitutive System for the Expression of hIL-22

All oligonucleotides and synthetic DNA blocks used in this study are listed in Supplementary Table 2. To generate pLOV33, the sequence of hIL-22 (codon optimized for *L. reuteri)*, was fused to the LAR_0089 signal peptide (SP000) and was synthesized by GenScript (NJ, USA). A NcoI restriction site (at the 5′ of the signal peptide) and an EcoRI site (at the 3′ of hIL-22) were also introduced in the sequence. Primers oLC_201 and oLC_689 and were used to amplify this sequence. The amplicon (insert) and the vector pSIP411 (a kind gift from Lars Axelsson, Nofima, Norway) were individually digested with NcoI and EcoRI (for 2 h at 37°C). The digestions were run on a 1% ultra-low melting point agarose (Invitrogen, CA, USA), and the products with the expected size were cut from the gel (backbone of pSIP411 of approximate 5.8 kb and the insert, 586 bp) and gel purified with the Wizard® Genomic DNA Purification Kit (Promega, WI, USA) according to the manufacturer's instructions. The DNA fragments were ligated with DNA ligase (NEB) at a 1:3 (vector:insert) molar ratio overnight at 16°C. Ligations were cleaned, precipitated with pellet paint, and transform into *E. coli* 1000 by electroporation. Sequence-verified plasmids were subsequently transformed into *L. reuteri* by electroporation as previously described.

To build pLS103 we used the Gibson cloning system, following the manufacturer's instructions. The signal peptide Lp_3050 from *L. plantarum* was codon optimized for *L. reuteri* 6475 and synthesized by IDT as a gblock (gLS103). gLS103 was used as template to amplify the insert by PCR using primers pLS103_2 and pLS103_5. The hIL-22 insert was amplified from pLOV33 using oligonucleotides pLS_103_3 and pLS103_6; whereas the backbone of the construct was amplified from pSIP411 with the primers pLS103_1 and pLS103_4. The fragments were mixed at a molar ratio of 1:1:1 and incubated with the Gibson Assembly® Master Mix (NEB, USA) for 4 h at 60°C. Similarly, pLS107 was built by amplifying the Usp45 sequence and the hIL-22 (gene codon optimized for *L. lactis*) from the gblock gLS107 with oligonucleotides pLS_107_2 and pLS_107_7, while the backbone was amplified from pNZ8048 with oligonucleotides pLS_107_1 and pLS_107_4. The reactions were assembled with the Gibson assembly mix, cleaned, and concentrated with pellet paint to be transformed into *E. coli* 1000 (as described for pLOV33). The plasmid was transformed into *Lactococcus lactis* as previously described (Zhang et al., 2009). Engineering of *L. casei* to produce hIL-22 was done by transforming pLOV33 into this strain as described for *L. reuteri*.

The vector for the expression of Elafin (pLOV002) was built as described for pLOV33. The sequence of human Elafin was synthesized by Blue Heron Biotech (Washington, USA) into the pUC minus backbone (pLOV101). The human Elafin sequence was PCR amplified with oligonucleotides oLC217 and oLC213, whereas the LAR_0089 signal peptide was amplified from *L. reuteri* 6475 genome with oligonucleotides oLC201 and oLC211. These fragments were fused by PCR, by mixing them to a 1:1 molar ratio, and then amplifying them with oligonucleotides oLC201 and oLC213 (which introduced the NcoRI and EcoRI restrictions sites at the 3′ and 5′ end, respectively). The PCR amplicon was cleaned with the Wizard® Genomic DNA Purification Kit (Promega, USA), digested with NcoI and EcoRI, from which the products were ligated in to the pSIP411 backbone, and confirmed as described for pLOV33.

### Transformation of *Lactobacillus* spp.

*L. reuteri* and *L. casei* were transformed by electroporation as previously described (Holo and Nes, 1989; Molin et al., 1992). Briefly, *Lactobacillus* cultures were grown to an OD_600_ between 0.7 and 0.9, and electroporated with one microgram of plasmid DNA. After transformation, cells were recovered for 3 h and then plated on media with the appropriate antibiotic selection. Bacteria are cured from their plasmids by growing them in broth without antibiotic for two generations, followed by replica plating on non-selective and selective MRS agar plates to identify colonies that lost the plasmid.

### Recombineering Experiments

To generate null mutants of proteases and amino-peptidases, we used recombineering as previously described (van Pijkeren and Britton, 2012). Briefly, *L. reuteri* LJO2 harboring pJP042 was grown to an OD_600_ between 0.55 and 0.65, then induced with 10 ng/ml of pSIP411 induction peptide for 20 min to promote the expression of RecT. Cultures were recovered for 3 h in one ml of MRS. Transformants that acquired the *rpo*B mutation targeted by the oJP577 oligomer were selected by plating the cells on MRS plates containing 25 μg/ml of rifampicin. Recombineering oligonucleotides used for this work (Supplementary Table 2) were ordered from IDT (USA) and the DNA was at a scale of 100 nm, desalted, without modifications. The oligonucleotide design and the screening were done as previously described, by van Pijkeren and Britton (2012).

### Western Blots for hIL-22

Expression and secretion of hIL-22 was analyzed by western blot. *L. reuteri* cultures were grown for 16 h and were sub- cultured in bMRS, and then induced with 50 ng/ml of pSIP411 induction peptide, when OD_600_ was between 0.9 and 1.0. Cultures were harvested after 5 h of induction. Supernatants were filtered sterilized (0.22 μm, low binding protein filter, [Millipore Sigma, USA]) and samples were used undiluted unless otherwise indicated. Cell pellets were re-suspended in 1:50 of the volume in lysis buffer (20 mM Tris–HCl, 10 mM EDTA, 1% Triton X-100, pH to 7.5) and disrupted with a MC table-top homogenizer (Constant systems Ltd, UK) at 30 KPSI (3x). The lysate was centrifuged at 4°C for 10 min at 20,000 × g, and the suspension was pipetted into a 4- 20% Criterion Tris-HCl gel (Bio-Rad) or 16.5% Tris-Tricine gel (Bio-Rad); along with 10 to 50 ng of recombinant hIL-22 (rhIL-22) (PeproTech, NJ, USA) as positive control. Supernatants and lysate samples were prepared with 2 × Laemmli buffer (Bio-Rad), boiled for 10 min, and then pipetted in to the protein gel according to the manufacturer's instructions. Protein gels were transferred onto a nitrocellulose membrane (Bio-Rad), and blocked with blocking solution (PBS, 5% Bovine Serum Albumin (BSA) and 0.05% Tween 20) for 1 h. The blot was probed with goat anti- hIL-22 antibody (R & D Systems, MN, USA) as the primary antibody at a 1:1000 dilution. Anti- goat HRP conjugated antibody (R& D Systems, MN, USA) was used as secondary antibody at a 1:1000 dilution. The antibodies were diluted and used as suggested by the manufacturer.

### Enzyme-Linked Immunosorbent Assay (ELISA)

ELISA was used to quantify the expression levels of hIL-22, human IL-10, and Reg3α. ELISA was performed using the Peprotech hIL-22 kit (Peprotech, NJ, USA), as recommended by the supplier. The standard curve was established from 31.25 to 1000 pg/ml using the standard curve interpolation function in GraphPad Prism using a second order polynomial function. The absorbance was measured at 415 nm with the Infinite F200 Microplate Reader (Tecan, Switzerland) Expression of IL-10 was measured from Colo205 cells-free of medium using the Human IL-22 Quantitative ELISA Kit (R & D Systems, USA) using the manufacturer's protocol. The standard curve was established from 15.62 to 2000 pg/ml using the standard curve interpolation function in GraphPad Prism using a second order polynomial function. The absorbance was analyzed at 415 nm with the Infinite F200 Microplate Reader (Tecan, Switzerland). Expression of Reg3α was measured from human J2 enteroids using the human Reg3α Quantitative ELISA Kit (R&D systems, USA) using the manufacturer's protocol. The standard curve was established from 15.6 to 1000 pg/mL using the standard curve interpolation function in GraphPad Prism using a second order polynomial function. The absorbance was analyzed at 450 nm with the Infinite F200 Microplate Reader.

### *In vitro* Bioactivity Assay in Colo205

To measure the ability of hIL-22 produced by *L. reuteri* to stimulate secretion of IL-10, we incubated supernatants of this strain with the Colo205 cell culture line. Cells were purchased from ATCC (ATCC® CCL-222TM) and grown in RPMI-1640 supplemented with 10% FBS at 37°C with 5% CO_2_, and maintained as suggested by the supplier (ATCC, VA, USA). Cells were seeded at a density of 2 × 10^5^ cells/well into 96 well cell culture plate. Treatment with recombinant hIL-22 (Peprotech Cat# 200-22) was used as a positive control, ranging from 400 pg/ml to 0.39 pg/ml. *L. reuteri* cultures were grown overnight at 37°C, and sub-cultured the next day in bMRS (EM 8 μg/ml) to an OD_600_ of 0.1. Cells were induced to express hIL-22 when the cultures reached an OD_600_ between 0.9 and 1.0 and incubated for 5 h. Subsequently, cultures were pelleted for 5 min at 3,000 × g and supernatants were filtered sterilized with a 0.22 μm, low binding protein filter (Millipore Sigma, USA). Supernatants were concentrated with an Amicon-10KDa Ultra-0.5 Centrifugal Filter (Millipore Sigma), for 10 min at 20,000 × g; followed by two fold serial dilutions 1:2 to 1:16. Supernatants were added to the pre-seeded Colo205 cultures at a 5% final volume (10 μl of 200 μl), and incubated at 37°C, 5% CO_2_ for 16 h. The cell free supernatant was used to analyze the expression of IL-10 by ELISA.

### Human Intestinal Enteroid Culture

Human jejunal intestinal enteroids (J2 lines) were obtained from the Baylor College of Medicine DDC Core in undifferentiated 3D cultures in Matrigel. Undifferentiated 3D cultures were maintained in high Wnt complete media with growth factors (CMGF+) and split 1:2 every 7–10 days depending on the density. CMGF+ consists of Advanced DMEM/F12 (Invitrogen), 100 U/mL penicillin-streptomycin (Invitrogen), 10 mM HEPES buffer (Invitrogen), and 1X GlutaMAX (Invitrogen) with 50% (v/v) Wnt3A-conditioned medium, 20% (v/v) R-spondin conditioned medium, 10% (v/v) Noggin-conditioned medium, 1X B-27 supplement (Invitrogen), 1X N-2 supplement (Invitrogen), 1 mM N-acetylcysteine (Sigma-Aldrich), 50 ng/mL mouse epidermal growth factor (EGF) (Invitrogen), 10 mM nicotinamide (Sigma-Aldrich, St. Louis, MO), 10 nM Leu-Gastrin I (Sigma-Aldrich), 500 nM A-83-01 (Tocris Bioscience), and 10 nM SB202190 (Sigma-Aldrich). In preparation for gene expression experiments, 3D cultures were laid in to confluent monolayers and maintained in CMGF+ supplemented with 10 μmol/L Y- 27632 initially for 2 days, and then subsequently in differentiation media supplemented with 10μmol/L Y027632 to induce monolayer differentiation. Differentiation medium consisted of the same components as CMGF+ without Wnt3A conditioned medium, R-spondin conditioned medium, SB202190, and nicotinamide and only 5% (v/v) Noggin conditioned medium. Differentiated monolayers were placed in CMGF- prior to use in gene expression experiments. *L. reuteri* cultures were grown overnight at 37°C, and sub-cultured the next day in bMRS (EM 8 μg/ml) to an OD_600_ of 0.1, cells were grown until an OD600 between 0.9 and 1.0 and subsequently pelleted and resuspended in *L. reuteri* cell culture media (LRCCM) containing mixture of 20% bMRS and 80% Roswell Park Memorial Institute medium (RPMI, containing 10% FBS). Cultures were then induced with 50ng/ml of pSIP411 induction peptide and incubated for 5 h. Subsequently, cultures were pelleted for 5 min at 3,000 × g and supernatants were filtered sterilized with a 0.22 μm, low binding protein filter (Millipore Sigma, USA). Supernatants were concentrated with an Amicon-10KDa Ultra-0.5 Centrifugal Filter (Millipore Sigma), for 10 min at 3,000 × g. Commercial recombinant hIL-22 (Peprotech Cat# 200-22) was used as a positive control. Bacterial supernatants were added to the monolayers at a 20% final volume (20 μl of 100 μl), and incubated at 37°C, 5% CO_2_ for 16 h. The cell free supernatant was used to analyze the expression of Reg3α by ELISA.

### Statistical Analysis

Student's *T*-test and ANOVA was used to determine whether differences among the groups were statistically significant (*p* < 0.05). Error bars indicate the standard deviation of the mean. All data analysis was performed using GraphPad Prism 7.0 software.

## Results

### Generation of an *L. reuteri* Strain to Efficiently Secret Active Human Interleukin-22 (hIL-22)

**Figure 1:**
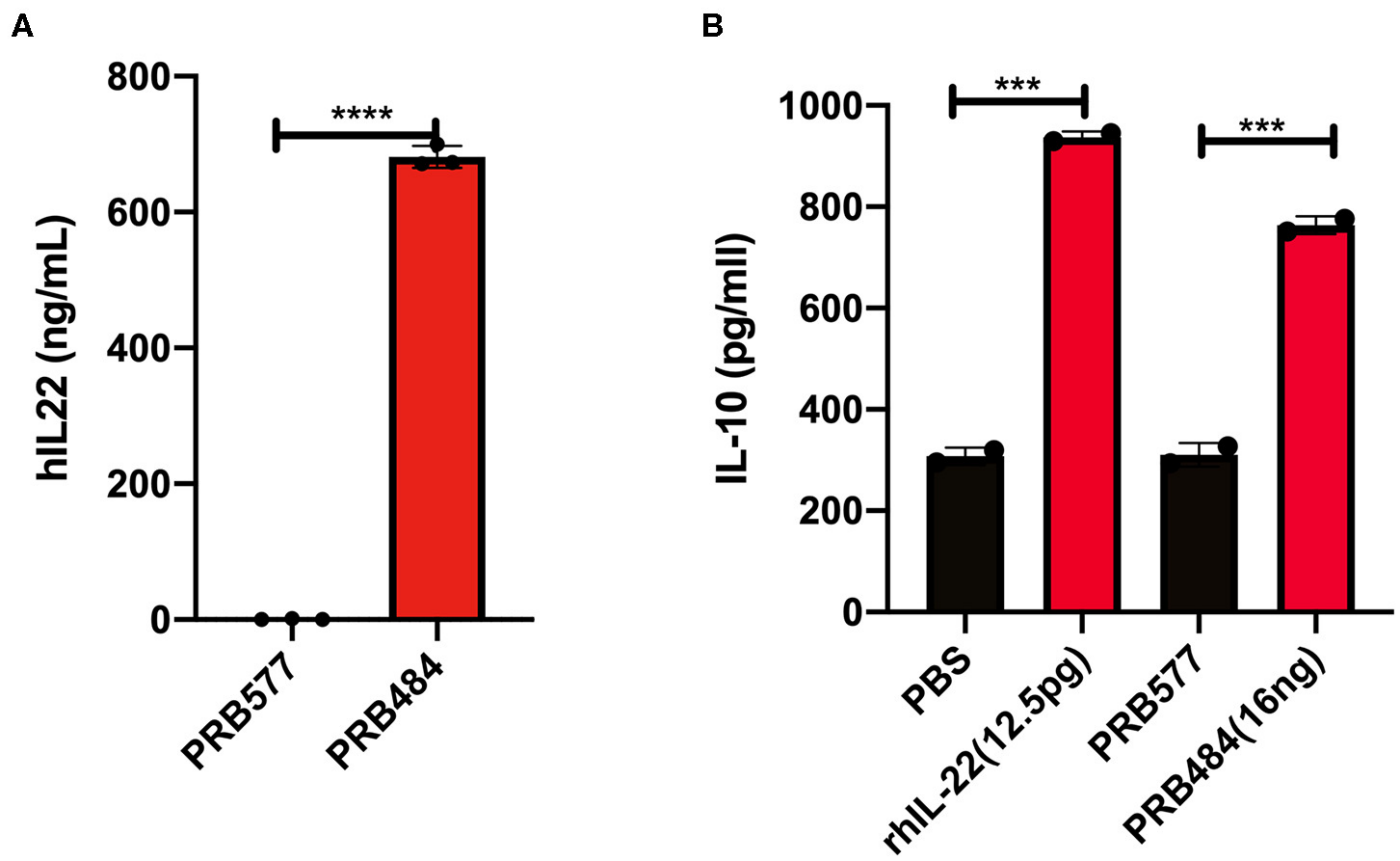
Expression and secretion of hIL-22 by *L. reuteri*. **(A)** Recombinant hIL-22 detected by ELISA in supernatants of recombinant *L. reuteri* (PRB484) upon induction. *L. reuteri* expressing pSIP411 was used as control (PRB577). **(B)** Induction of IL-10 production from Colo205 cells upon treatment with commercial rhIL-22 as compared to bacterially secreted IL-22. Data are mean of *n* = 3 with error bars representing deviation from the mean; comparisons performed with *t*-tests (two groups) or analysis of variance (ANOVA) (multiple groups). ***P < 0.001, ****P < 0.0001.

We aimed to engineer *L. reuteri* 6475 to efficiently produce and secrete active hIL-22. For this purpose, we initially constructed an inducible system based on the pheromone-inducible gene expression system pSIP411, to promote inducible secretion of hIL-22 (Sorvig et al., 2005). We fused the codon-optimized sequence of hIL-22 to the signal peptide of the LAR_0089 gene in *L. reuteri* F275 (PRB484), generating the vector pLOV33. Although this signal peptide has not previously been explored for the purpose of secretion of protein in *L. reuteri*, we selected it because it harbors a motif that efficiently drives secretion of proteins in the gram-positive bacteria Streptococcus pneumonia (YSIRK motif) (Bae and Schneewind, 2003). To evaluate the ability of *L. reuteri* to promote secretion of hIL-22 efficiently, we analyzed the levels of hIL-22 in supernatants of the strain engineered to secrete this cytokine. As expected, hIL-22 was detected in supernatants of PRB484 at concentrations of ~700 ng/mL, but it was undetected in uninduced conditions (Figure 1A).

### Biological Activity of hIL-22 Produced by *L. reuteri*

We subsequently assessed the biological activity of hIL-22 secreted by PRB484. The production of IL-10 and the phosphorylation of STAT3 (pSTAT3) in the cell culture line Colo205 in the presence of hIL-22 are commonly used assays to demonstrate the activity of this protein (Nagalakshmi et al., 2004; Loera-Arias et al., 2014; Lin et al., 2017). To investigate if hIL-22 secreted by *L. reuteri* 6475 was biologically active, we incubated Colo205 cells with commercial recombinant hIL-22 (rhIL-22), supernatants from PBR484 or *L. reuteri* harboring pSIP411 as a negative control (PRB577). Colo205 cells incubated with 12.5 pg/ml of rhIL-22 produced ~900 pg/ml of IL-10, similar to the levels induced by supernatants from PRB484 (Figure 1B), which secretes hIL-22 at a concentration of ~700 ng/mL. On the other hand, supernatants from PRB577 induced the same levels of IL-10 as media alone, demonstrating that stimulation of IL-10 is only achieved in the presence of rhIL-22 positive control or hIL-22 produced by *L. reuteri*. These results show that the hIL-22 produced by *L. reuteri* is biologically active, though with significantly decreased efficacy as compared to commercial recombinant hIL-22. In order to achieve a similar level of IL-10 production as achieved by 12.5 pg/mL of commercial rhIL-22, we needed to add ~1,000 times (16 ng vs. 12.5 pg, respectively) more hIL-22 produced by *L. reuteri*. This suggests that only about 0.1–1% of the total hIL-22 protein secreted in supernatants was biologically active (Figure 1B).

**Figure 2:**
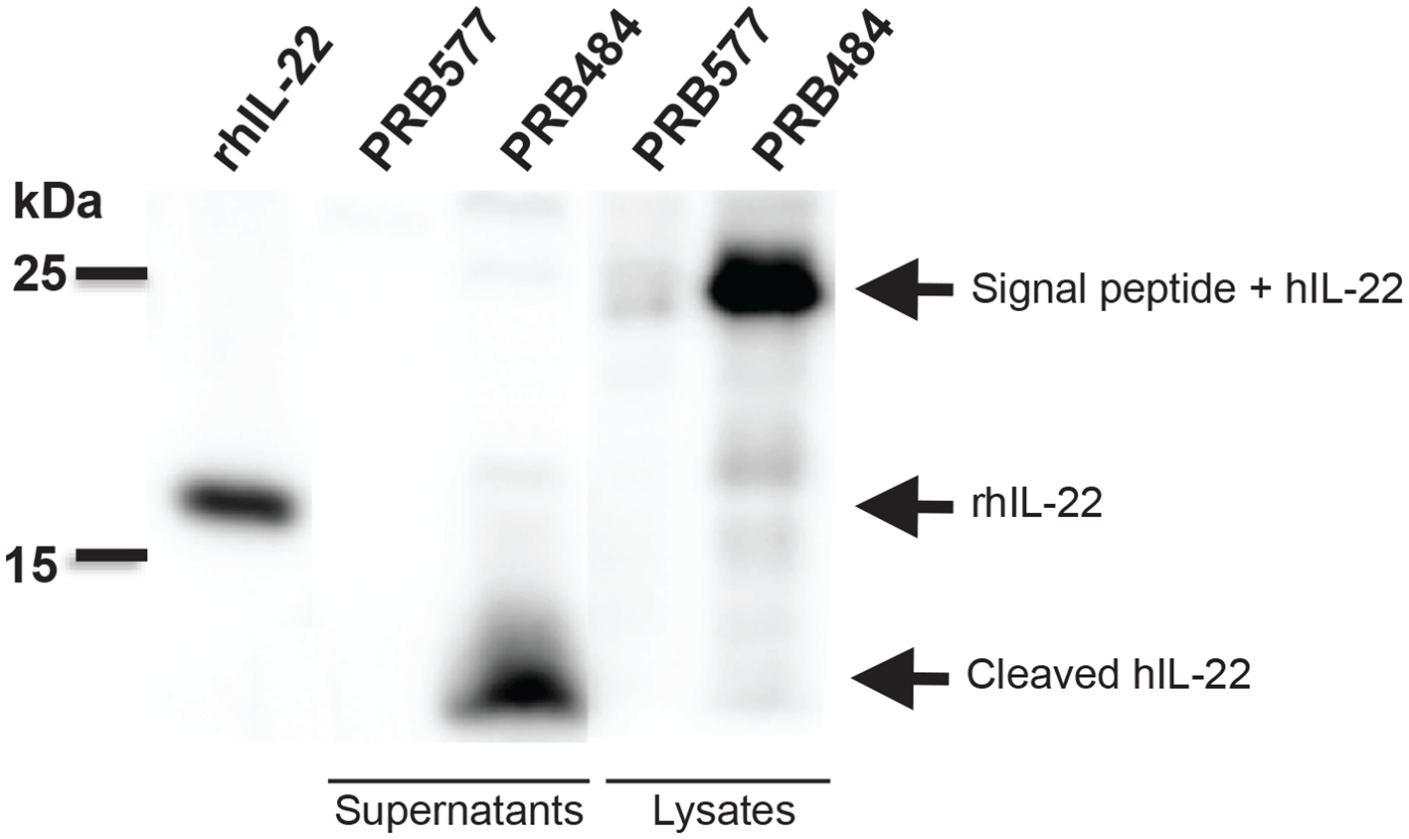
Bacterially secreted hIL-22 is cleaved. Western blot of induced bacterial supernatant and cell lysates. Cell lysates of PRB484 show a band at ~23 kDa, the expected size for hIL-22 fused to the signal peptide. In comparison, supernatants from induced PRB484 cultures show protein bands much smaller in size, ~4 kDa representing cleaved hIL-22.

To investigate the reason for the decreased activity of *L. reuteri* secreted hIL-22 as compared to commercial recombinant hIL-22, we looked at the processing of this cytokine in supernatants and cell lysates of hIL-22 secreting *L. reuteri* (Figure 2). On western blot, cell lysates of PRB484 showed a band at ~23 kDa, the expected size for hIL-22 fused to the signal peptide. In comparison, supernatants from induced PRB484 cultures showed protein bands with sizes ranging from 4 to 10 kDa, instead of the expected size of hIL-22 (16 kDa). No bands were observed in supernatants or lysates of PRB577. These findings demonstrated that although *L. reuteri* adequately expresses hIL-22 intracellularly, the vast majority of the protein is being cleaved as it is translocated through the membrane or when it reaches the extracellular environment, thus inactivating the much of the secreted protein.

### hIL-22 Is Also Cleaved in Other Lactobacilli Engineered to Secrete hIL-22

**Figure 3:**
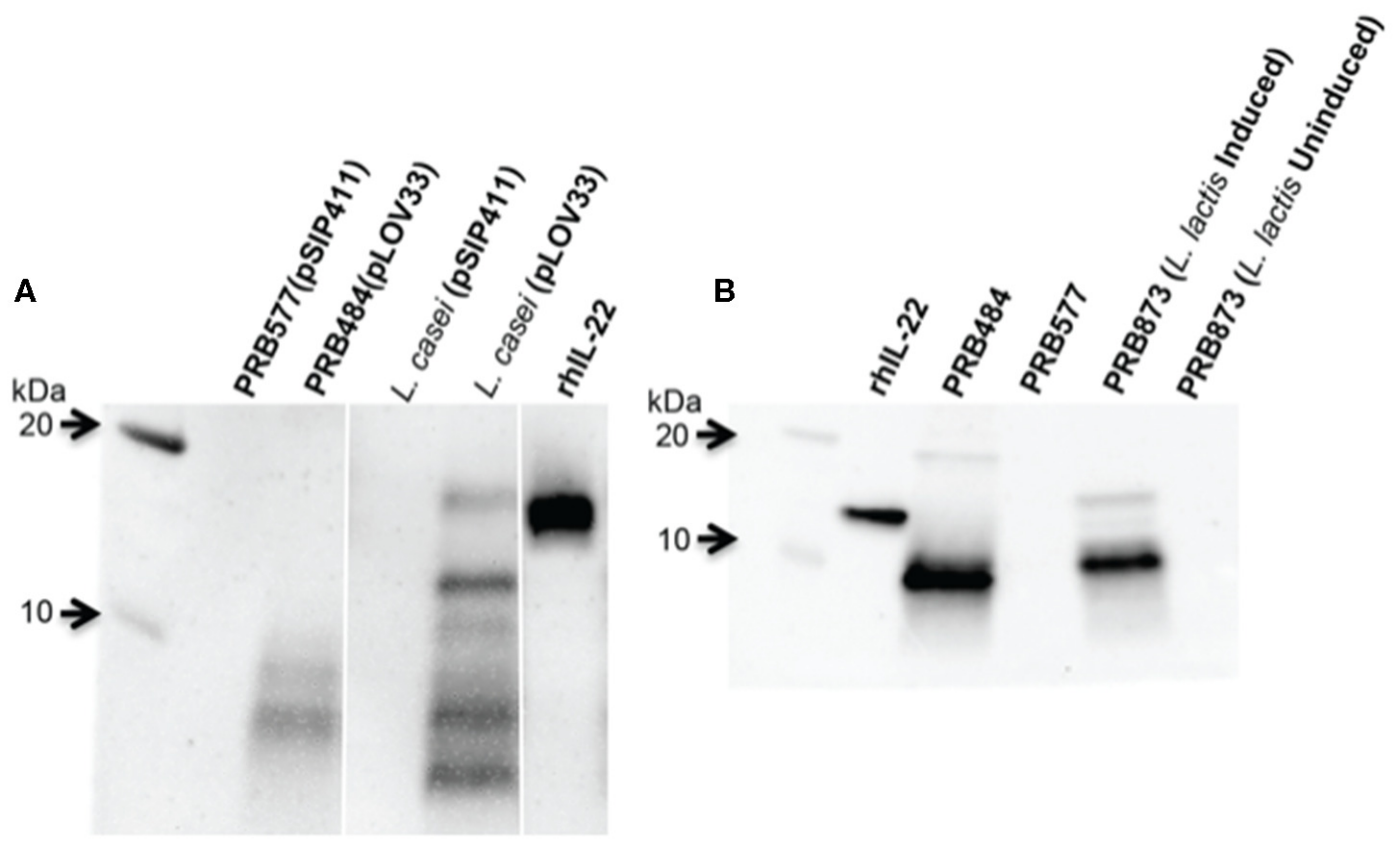
hIL-22 is also cleaved by other Lactobacilli engineered to secrete hIL-22. **(A)** Western blot of hIL-22 from supernatants of *L. casei* harboring pLOV33 or pSIP411 showing hIL-22 secreted by *L. casei* is cleaved similar to L. reuteri. **(B)** Western blot of hIL-22 from supernatants of *L. lactis* harboring pLOV33 (PRB873) showing cleavage of IL-22.

In order to test if this cleavage was specific to *L. reuteri*, we engineered other LAB including *L. lactis* and *L. casei* to secrete hIL-22. We demonstrated that degradation of hIL-22 was not an exclusive response displayed by *L. reuteri* since other LAB, such as *L. lactis* and *L. casei* also cleaved hIL-22 expressed by our construct (Figure 3). In contrast, we were also able to engineer *L. reuteri* to secrete Elafin, also known as peptidase inhibitor 3 (PRB841). This protein has previously been shown to be secreted appropriately by *L. lactis* (Motta et al., 2012). Western blot of cell lysates and supernatants of *L. reuteri* PRB841 cells secreting Elafin produced a unique protein band of about 17 and 14 kDa, respectively; when analyzed with either monoclonal or polyclonal antibodies (Supplementary Figure 1). These protein bands exhibited the expected sizes for the precursor (with the signal peptide) and the secreted form of Elafin, demonstrating that Elafin is not subject to proteolysis by *L. reuteri* 6475. These results show that *L. reuteri* does not cleave all heterologous proteins secreted to the extracellular medium, but rather it is a response linked to the secretion of hIL-22 itself.

### Attempts to Improve the Levels of Active hIL-22 Secreted by *L. reuteri*

We explored several strategies to prevent hIL-22 degradation, including the use of protease inhibitors and the mutation of membrane-associated proteases (Supplementary Figures 2, 3). Incubation of commercial rhIL-22 with either supernatant from *L. reuteri* harboring a control vector (PRB577) or cultures of this strain (cells and supernatants) did not display any degradation of rhIL-22, suggesting that cleavage of IL-22 was not directly caused by an extracellular protease (Supplementary Figure 2).

Some *Lactobacillus* strains target and show high proteolytic activity toward peptides that have prolines at the N-terminus (Duar et al., 2015). Since the second amino acid of hIL-22 is a proline, we hypothesized that this amino acid plays a vital role in cleavage of the hIL-22 secreted by *L. reuteri*. We therefore created a P2G mutation, a proline to glycine amino acid change, which has similar properties to proline, in order to evaluate the effect that this amino acid substitution has on hIL-22 proteolysis (PRB748). We also built a double mutant of the first and second proline present in the hIL-22 sequence (ΔP2GΔP17G) (PRB848). Based on western blot assays, the double proline mutant yields more hIL-22 than the single proline mutant (Supplementary Figure 4A). However, measurements of IL-10 produced by Colo205 cells incubated with supernatants of PRB848 demonstrated that the biological activity of hIL-22 is completely abolished when both prolines are mutated; whereas PRB748 has about 4–6 times more activity than the strain expressing the wild-type sequence of hIL-22 (PRB484) (Supplementary Figure 4B). These results show that the mutation of the first proline (P2G) at the N-terminus of hIL-22 elicits an incremental improvement in the expression of active hIL-22. Nonetheless, neither visibly improved processing of this protein as observed by western blot.

**Figure 4:**
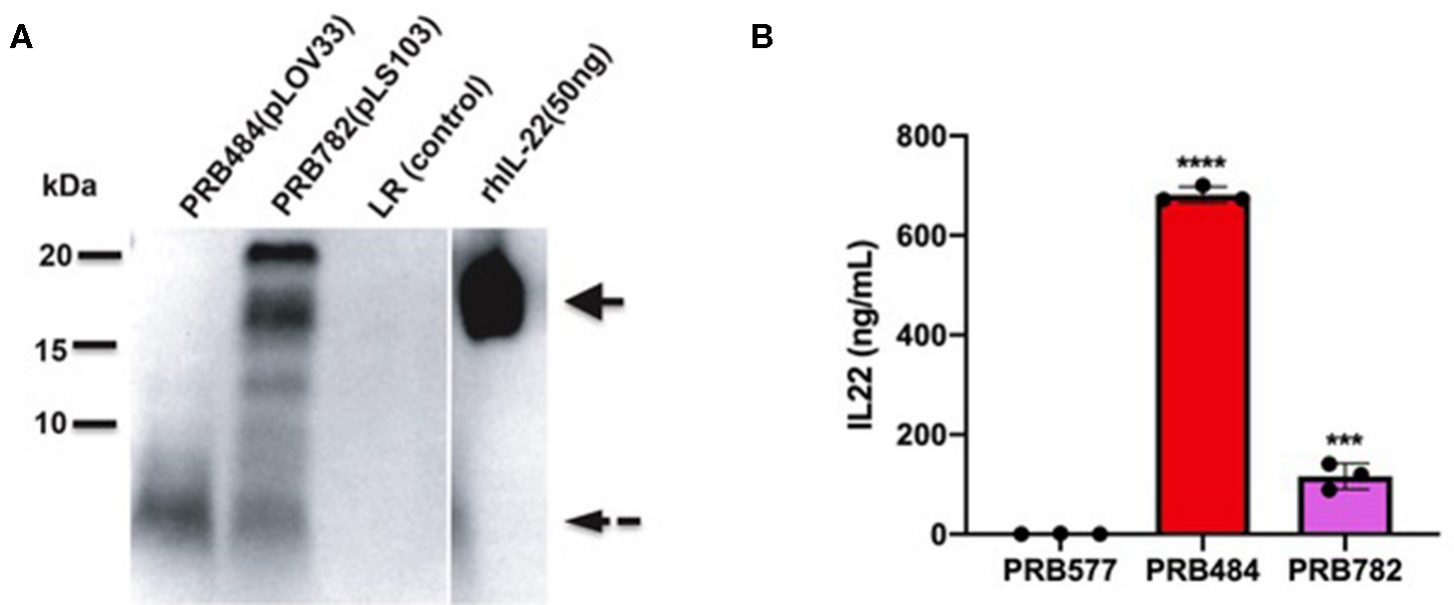
PRB782 shows improved processing of secreted hIL-22. **(A)** Western blot of bacterial supernatant from PRB782, PRB484, and PRB577 shows new strain of recombinant *L. reuteri* (PRB782) with production of more intact hIL-22. **(B)** hIL-22 ELISA showing lower concentrations of secreted hIL-22 from PRB782 as compared to PRB484. Data are mean of *n* = 3 with error bars representing deviation from the mean; comparisons performed with *t*-tests (two groups) or analysis of variance (ANOVA) (multiple groups). ***P < 0.001, ****P < 0.0001.

**Figure 5:**
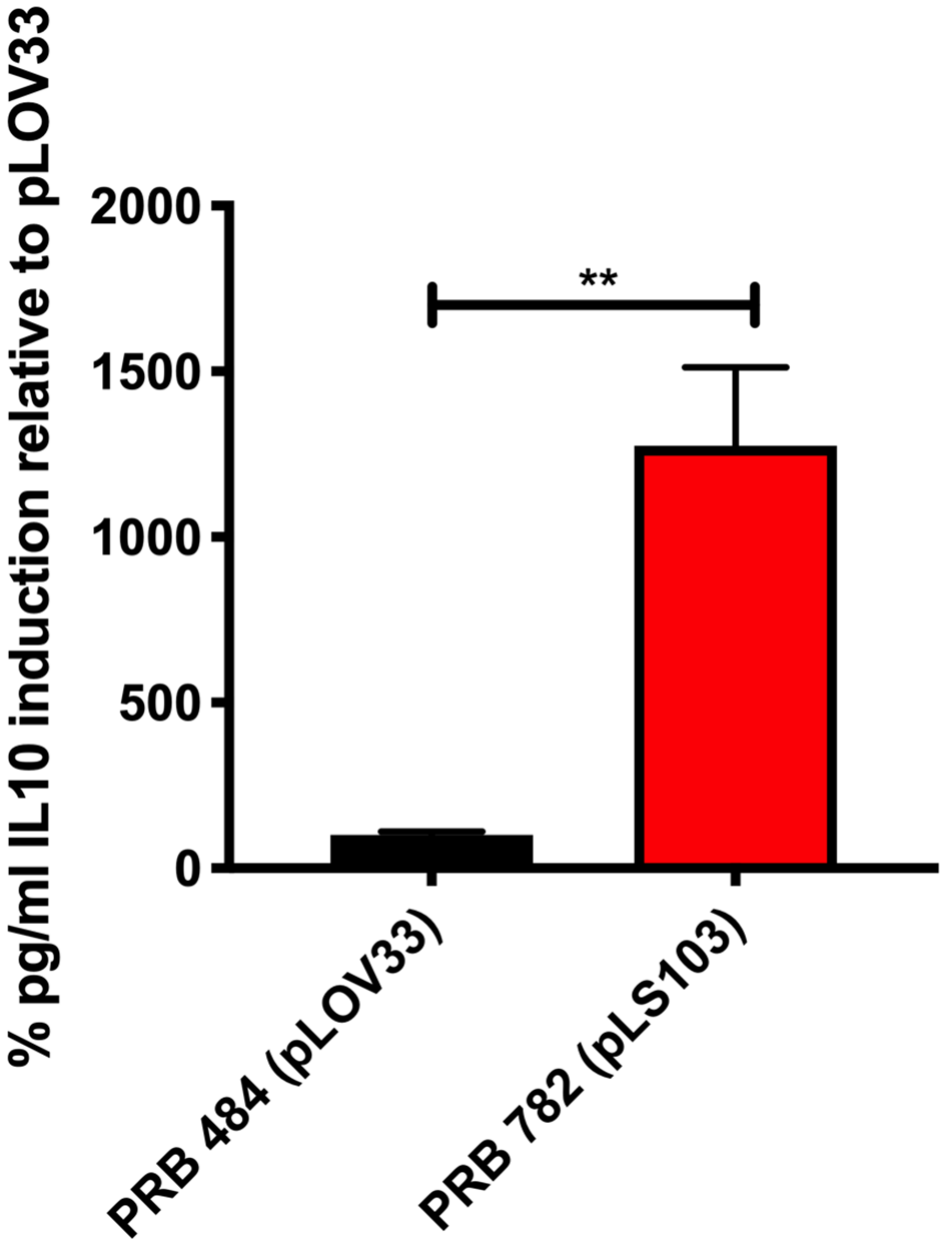
PRB782 shows improved biological activity. Induction of IL-10 production with treatment of Colo205 cells with induced bacterial supernatant from PRB484 and PRB782 showed PRB782 to have significantly (~15-fold) higher biological activity as compared to PRB484. Data represent the average of 3 biological replicates with error bars indicating standard deviation from the mean. Comparison performed with *t*-tests. **P < 0.05.

Finally, we tested several different signal peptides to identify if any promoted secretion of full length hIL-22. Through this strategy, we were able to find one *L. reuteri* strain (PRB782) that significantly improved processing of hIL-22 based on western blot assays (Figure 4). In this strain, the original signal peptide was replaced for the signal peptide from the Lp_3050 gene from *L. plantarum* (plasmid named pLS103), which has previously shown to promote efficient secretion of recombinant proteins in *Lactobacillus* spp (Karlskas et al., 2014). PRB782 not only improved processing of hIL-22 but it also increased the overall amount of biologically active protein. Incubation of Colo205 cells with supernatants from this strain demonstrated that *L. reuteri* PRB782 produces about 10–20 times more biologically active hIL-22 than the strain that PRB484 (Figure 5). However, the overall production of hIL-22 by PRB782 is ~150 ng/ml, five times lower than PRB484 (Figure 4).

### Production of hIL-22 Affects the Growth of *L. reuteri*

**Figure 6:**
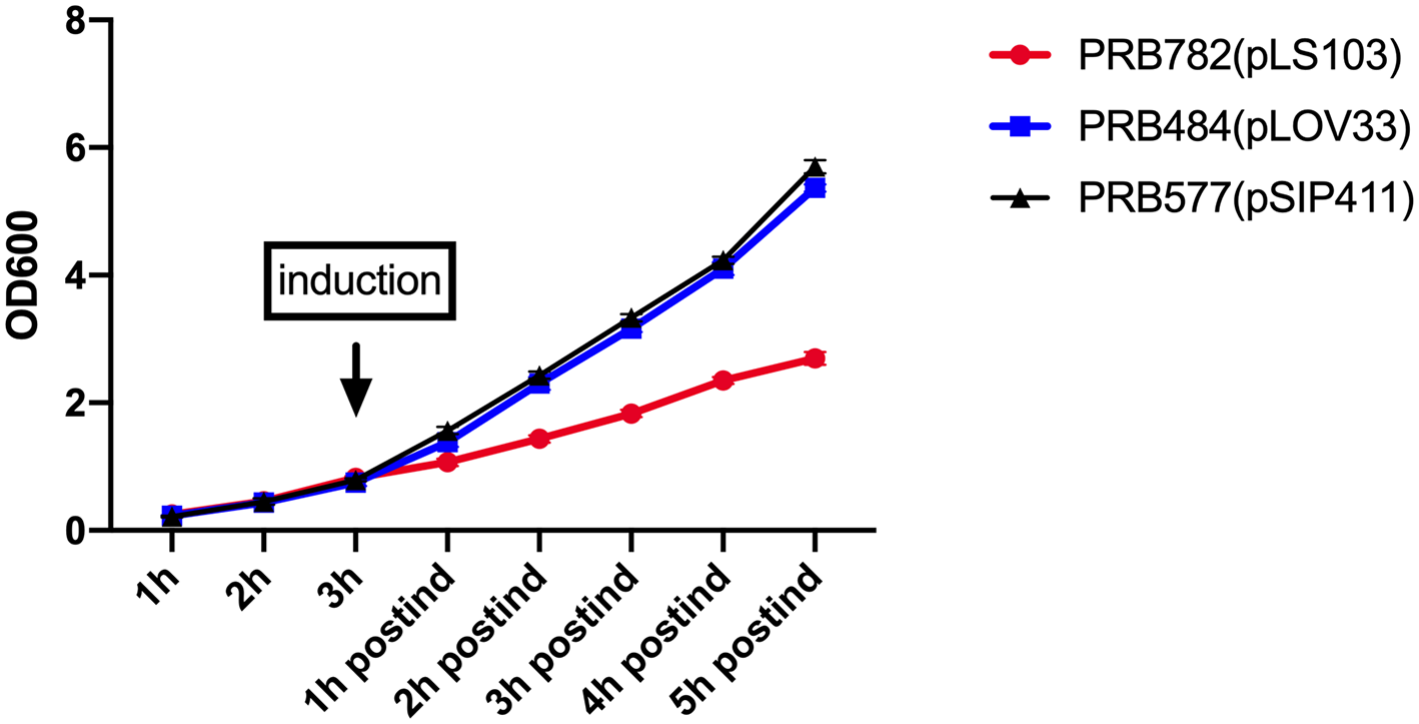
Production of hIL-22 affects the growth of *L. reuteri*. OD_600_ measurements of PRB782, PRB484, and PRB577 before and after induction of hIL-22. Data represent the average of 3 biological replicates.

To examine the effect of induced secretion of hIL-22 on the growth of recombinant *L. reuteri*, we measured OD_600_ of strains both before and after culture induction (Figure 6). Interestingly, growth curves of our strains during the production of hIL-22 demonstrated that expression of this protein has a profound negative impact on the growth of PRB782, but not on PRB484, suggesting that the secretion of active hIL-22 may be toxic to *L. reuteri*. Therefore, although PRB782 considerably improved processing of hIL-22 and the overall amount of active protein; the growth of this strain is extremely impaired when protein production is induced. This severe growth defect was also observed in strains engineered to constitutively express hIL-22, which generated abnormal and inactive forms of hIL-22 in supernatants (data not shown). Taken together this data suggests that the production of the active form of hIL-22 is toxic to *L. reuteri* and thus may be acting as a selective pressure against the generation of a strain of *L. reuteri* with efficient secretion of hIL-22.

### hIL-22 Secreted by PRB782 Triggers the Production of Antimicrobial Peptides in Human Enteroids

**Figure 7:**
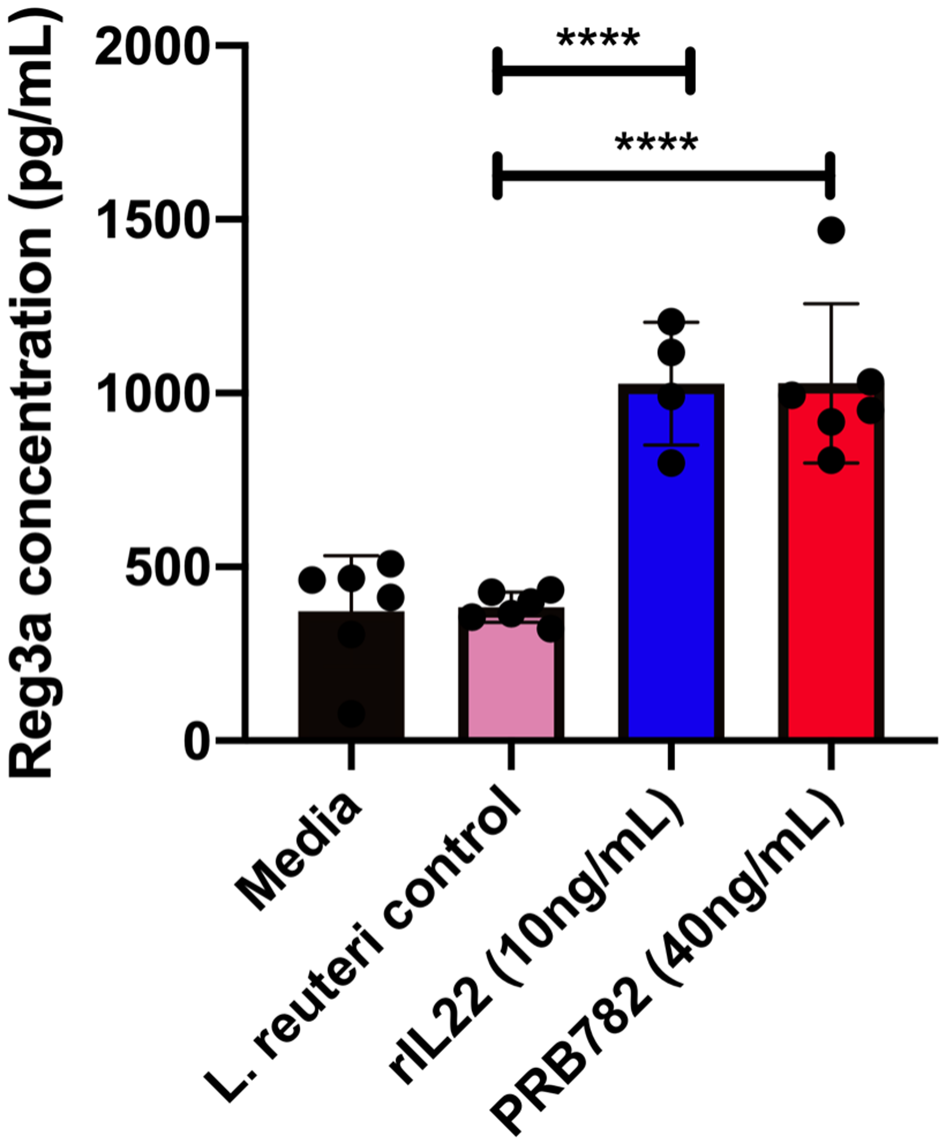
hIL-22 secreted by PRB782 induces production of the antimicrobial peptide Reg3α in human enteroids. Reg3α present in enteroids supernatants treated with PRB484, PRB782, and pRB577. Monolayer of enteroids were treated for 16 h and detection of Reg3α was done by ELISA. Data represent the average of six biological replicates. Error bars indicate the standard deviation of the mean. Comparisons performed with analysis of variance (ANOVA) (multiple groups). ****P < 0.0001.

To validate that the hIL-22 produced by *L. reuteri* was capable of driving the production of antimicrobial peptides in the context of a multicellular intestinal tissue, we investigated the ability of hIL-22 to drive the production of regenerating islet derived protein 3 alpha (Reg3α) in human intestinal enteroids. Reg3α plays a vital role in innate immunity and the antimicrobial response within the human intestine and has previously been shown to be induced by hIL-22. We incubated human enteroids derived from jejunum with rhIL-22, supernatants from PRB782 or the control strain PRB577. Enteroids treated with PRB782 expressed significantly higher levels of the Reg3α by ELISA compared to cells treated with PRB577 (Figure 7). These results further demonstrate that the hIL-22 produced by *L. reuteri* is biologically active and can trigger the production of antimicrobial peptides in human cells, which is a crucial component of the therapeutic potential that hIL-22 has for the maintenance of the intestinal barrier.

## Discussion

In this study we explored the ability of *L. reuteri* to express and secrete active hIL-22 at high levels. Previous reports of lactobacilli expressing active IL-22 (either human or murine) have yielded functional proteins with a wide variety of expression levels of human (*L. lactis*, 2–20 ng/ml) or mouse IL-22 (*L. plantarum*, 500 ng/ml). We found that expression and secretion of hIL-22 provided a fitness cost to *L. reuteri* that resulted in much of the protein being cleaved upon secretion. We identified that this cleavage is not a specific effect of *L. reuteri* since both *L. lactis* and *L. casei* strains also cleaved most of the hIL-22 secreted from these strains.

Why expression of functional hIL-22 from lactobacilli causes a severe growth defect is still unclear. IL-22 regulates host production of antimicrobial peptides and thus has been thought of as an antimicrobial mediator. One possibility is that secreted hIL-22 is directly mediating the growth inhibition, however to date there has been no report of IL-22 possessing antimicrobial activity of its own. Preliminary experiments in our lab did not uncover any significant antimicrobial activity of purified hIL-22. Another possibility is that the high level of expression of hIL-22 is creating strain on the expression and secretion systems of *L. reuteri*, leading to growth defects. Interestingly, secretion of mIL-22 from *L. reuteri* also resulted in large scale cleavage of mIL-22 (despite the use of a different signal peptide), similar to our findings in this study (JP van Pijkeren, personal communication). If this is the case, to mitigate the detrimental effects of hIL-22 secretion by *L. reuteri*, alternate expression systems that would allow for controlled expression of hIL-22 in the gut, such as through the use of anaerobic promoters, may further improve therapeutic delivery within the intestinal lumen.

Despite our initial setbacks, we found significant hIL-22 expression in the supernatant of our constructs, which allowed us to pursue optimization of the activity. Previous groups have shown the ability to produce IL-22 and we suspect they had similar problems with IL-22 toxicity that we observed in our study. Expression of hIL-22 from *L. lactis* (2–20 ng/ml) showed 50–500 fold less IL-22 protein than what we report here as well as mIL-22 (*L. plantarum*, 500 ng/ml) (Loera-Arias et al., 2014; Lin et al., 2017). The low levels of hIL-22 production in *L. lactis* and our work showing cleavage of hIL-22 in *L. lactis* when hIL-22 levels are much higher suggest that hIL-22 also has a negative impact on *L. lactis*. Similarly, the similar production levels of IL-22 in *L. paracasei* were associated with ~60% of the cells losing the plasmid in an overnight culture, supporting that IL-22 production provides a fitness cost in lactobacilli.

Since intracellular hIL-22 is not degraded and commercial recombinant hIL-22 is not cleaved when incubated with supernatants or *L. reuteri* cells, we hypothesize that degradation of hIL-22 occurs either during signal peptide processing or translocation of hIL-22 to the extracellular medium. Analysis by mass spectrometry of the proteolytic products present in supernatants revealed that multiple proteolytic products are generated after cleavage of hIL-22, suggesting that this is a complex process carried out by several proteases during secretion. Processing of secreted hIL-22 was able to be significantly improved by changing the existing signal peptide for one (Lp_3050) from *L. plantarum*. Alternatively, hIL-22 may be mediating a direct antimicrobial effect on *L. reuteri*. These results highlight the importance of the signal peptide in the production and secretion of proteins from *L. reuteri*. Understanding this effect can lead to the development of strategies that maximize the use of *L. reuteri* as a microbial cell factory.

Previous work on creating bacterial delivery systems for the secretion of human and mouse IL-22 have mainly tested *in vitro* activity by measuring the production of IL-10 from Colo205 cells (Loera-Arias et al., 2014; Lin et al., 2017). This assay, however, might not necessarily reflect the ability of hIL-22 to strengthen mucosal immunity at epithelial surfaces and promote tissue repair, effects that are essential to achieving its therapeutic effect *in vivo*. We demonstrated that hIL-22 secreted by *L. reuteri* (PRB782) is not only able to enable secretion of IL-10 in Colo205 cells, but also able to stimulate production of the antimicrobial peptide Reg3α, a biomarker for intestinal GVHD, in human intestinal enteroids. Human intestinal enteroids are cultures derived from the intestinal crypts of human intestinal biopsies and have advantages over traditional cell culture lines since they are non-cancerous, non-transformed, and are able to be differentiated into all of the cell types of the intestinal epithelium. The efficacy of our secreted hIl-22 on these intestinal enteroid cultures show promise that our secreted cytokine will be active on human intestinal epithelium *in vivo*.

Previous work looking at the effects of IL-22 on mouse intestinal enteroids have shown IL-22 to have a direct effect on the intestinal stem cell (ISC) niche, possibly through the expansion of transit amplifying cells that exist within the intestinal crypt (Lindemans et al., 2015; Zha et al., 2019; Zhang et al., 2019; Zwarycz et al., 2019). IL-22 has also been shown to act on Paneth cells and promote the secretion of antimicrobial peptides within mouse intestinal enteroids which further helps to maintain the ISC environment (Lindemans et al., 2015). All of these findings suggest that IL-22 may play an important role in mediating epithelial regeneration. Special interest has developed in the use of IL-22 in therapy for intestinal diseases such as GVHD and IBD. Our work shows that bacterially secreted hIL-22 can similarly act upon human intestinal enteroids and supports the idea of *L. reuteri* as a therapeutic delivery system for hIL-22 in treatment of human disease.

Moving forward with this work, we will be looking to test our engineered *L. reuteri* within an *in vivo* animal model of disease. In order to achieve success, we will need to optimize our delivery system to maximize production of functional protein in addition to being able to function within an anaerobic environment that is dynamic. The use of promoters exclusively induced in anaerobic conditions could be explored as a strategy to maximize protein production in the GIT and mitigate the toxic effect of hIL-22 on *L. reuteri* until delivery. Additionally, the stabilization of hIL-22 by integration into the chromosome, could significantly improve the likelihood of delivering an increased and constant amount of active hIL-22 to the GIT. An alternative strategy of delivering proteins that are not efficiently secreted would be through the use of phages to naturally induce *L. reuteri* lysis and subsequently protein delivery, as was recently shown in a mouse model of ethanol induced liver disease (Hendrikx et al., 2019).

## Conclusions

Engineered probiotics or commensal bacteria are an innovative approach for the controlled delivery and *in situ* production of complex molecules and therapeutics. hIL-22 is a cytokine with promising therapeutic value that can be utilized to treat pathologies where the integrity of the intestinal barrier is disrupted, such as in GVHD or IBD. Here we sought to demonstrate that *L. reuteri* 6475 can be engineered to secrete active hIL-22. After exploring several strategies to optimize *L. reuteri* secretion of hIL-22, we generated a strain (PRB782) that was capable of secreting high amounts of biologically active hIL-22. Furthermore, we have shown that this hIL-22 secreted by *L. reuteri* was capable of promoting production of Reg3α in human intestinal enteroids, demonstrating the potential of this engineered strain of bacteria as a therapeutic alternative for the delivery of hIL-22 to the GIT.

## Data Availability Statement

The datasets generated for this study are available on request to the corresponding author.

## Author Contributions

LO-V, AG, and RB: concept and design. LO-V, AG, LS, and RB: intellectual contribution. LO-V, AG, and LS: data acquisition. AG, LO-V, LS, and RB: data analysis, statistical analysis, and interpretation. AG, LO-V, and RB: drafting and editing manuscript. RB: obtained funding.

## Conflict of Interest

The authors declare that the research was conducted in the absence of any commercial or financial relationships that could be construed as a potential conflict of interest.
