# Supplementary material for: Challenges and Pitfalls in the Engineering of Human Interleukin 22 (hIL-22) Secreting Lactobacillus reuteri
Source: Front Bioeng Biotechnol. 2020 Jun 5;8:543. doi: 10.3389/fbioe.2020.00543 (PMC7289926; doi:10.3389/fbioe.2020.00543)
Supplement: Supplementary file 1 [file Data_Sheet_1.pdf]

SUPPLEMENTARY FIGURES

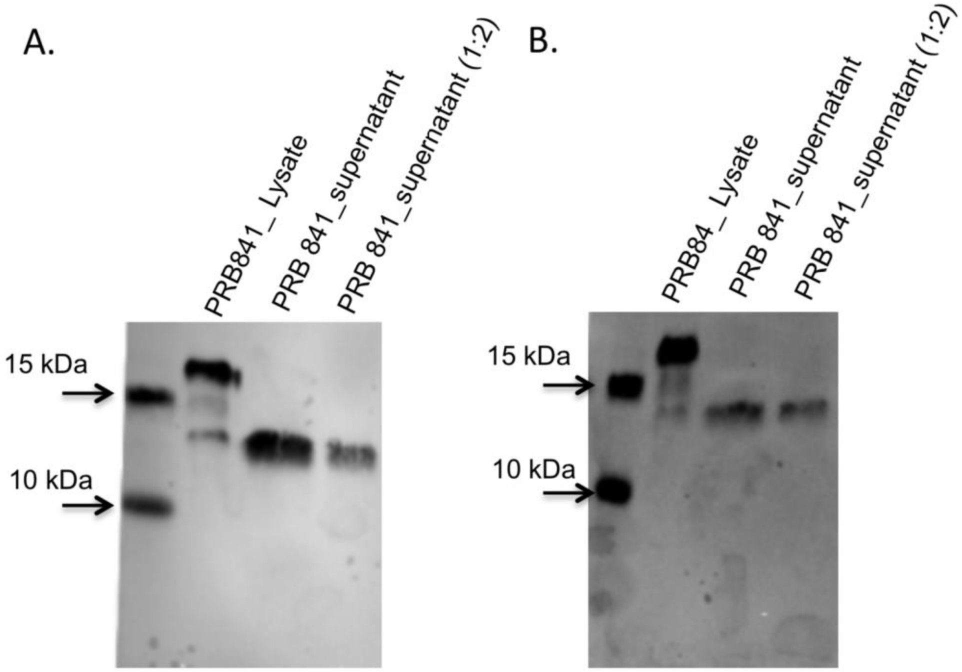

Supplementary Figure 1. *L. reuteri* does not cleave secreted Elafin. Western blots of the expression of Elafin in supernatants or cell lysates of recombinant *L. reuteri* (PRB 841), detected with a monoclonal antibody (A) or a polyclonal antibody (B) both diluted (1:2) or undiluted.

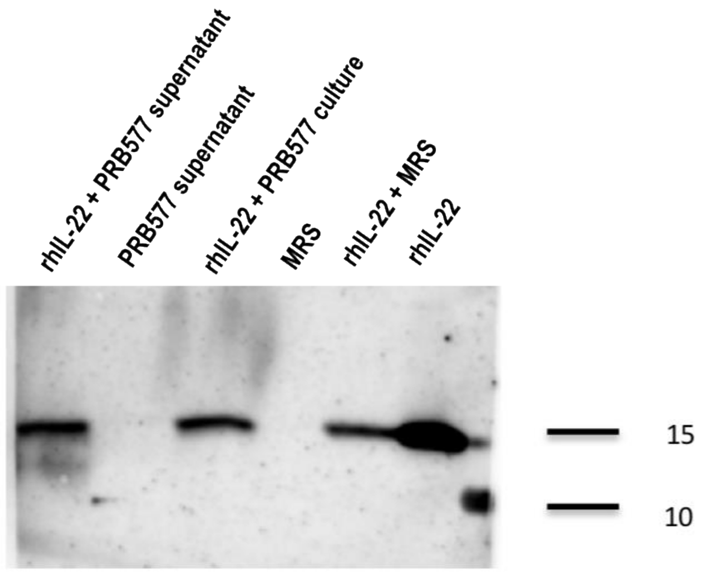

Supplementary Figure 2. Incubation of commercial rhIL-22 with bacterial supernatants does not cause cleavage of protein. Western blot of commercial hIL-22 incubated with bacterial supernatant, culture, and bacterial media shows no cleavage of IL22 suggesting cleavage of IL-22 was not directly caused by an extracellular protease.

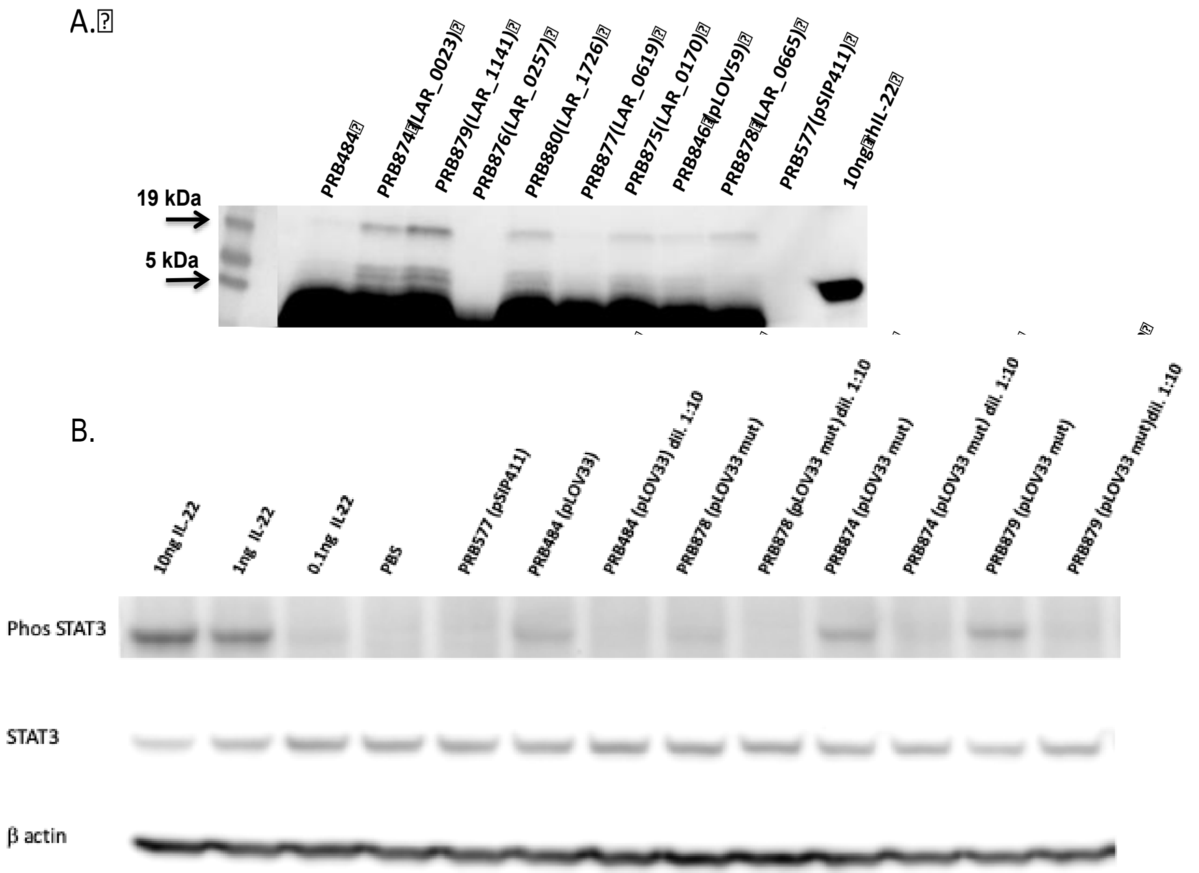

Supplementary Figure 3. Mutations of proteases improve processing of hIL-22 in PRB484. (A). Western blot of hIL-22 on supernatants of PRB484 or null mutants of predicted proteases that were screened for high production of hIL-22. The null mutants expressed hIL-22 from pLOV33. PRB577 was used as negative control and commercial hIL-22 as positive control (rhIL-22). (B) Biological activity of hIL-22 secreted by PRB484 or null mutants of proteases: PRB874, PRB878, PRB879. pSTAT3 expression from HT29 cells incubated with rhIL-22 or *L. reuteri* supernatants was measured by western blot. PBS and PRB577 were used as negative control. Supernatants loaded as undiluted or diluted (1:10). STAT3 and β- actin were used as internal controls.

A. B.

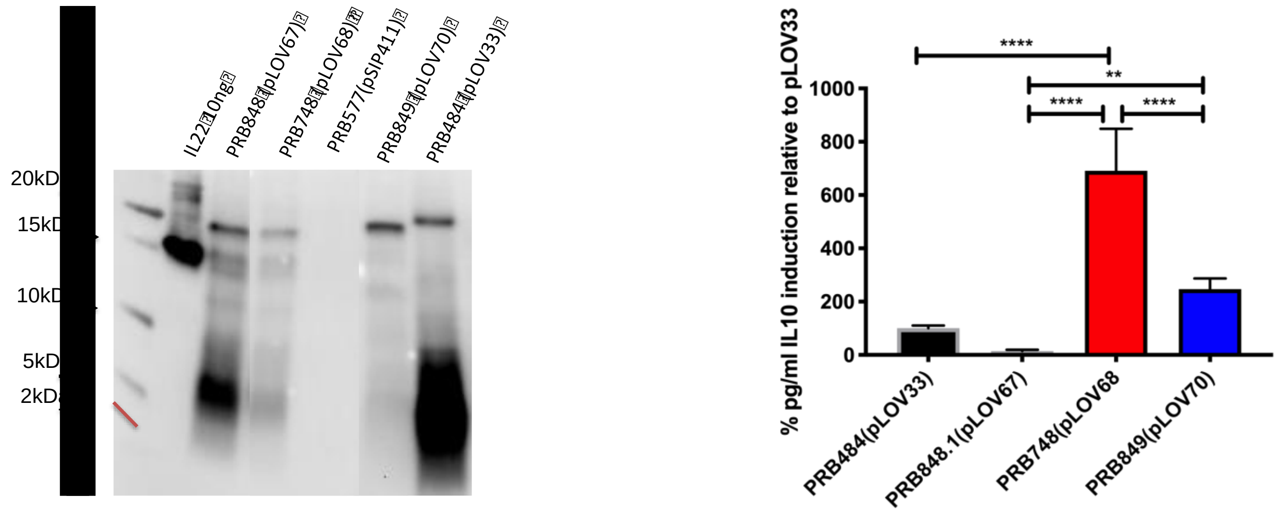

Supplementary Figure 4. Mutation of first proline in the N terminus of hIL-22 improves bioactivity of secreted protein. A. Western blot showing expression of hIL-22 in which either one proline (PRB748) or both N terminus prolines (PRB848) were mutated. An *L. reuteri* strain expressing a pro-peptide at the N term (PRB849) was also evaluated. B. IL10 ELISA showing biological activity of bacterially secreted hIL-22 from proline mutated strains on Colo205 cell cultures.

Supplementary Table 1. Additional strains created in this work.

Strain	Description	Source
PRB841	*L. reuteri* harboring pLOV02 for inducible expression of Elafin	This work
PRB848	*L. reuteri* harboring pLOV67 (double N terminal proline mutations)	This work
PRB748	*L. reuteri* harboring pLOV68 (single N terminal proline mutation)	This work
PRB849	*L. reuteri* harboring pLOV70 (pro-peptide added to N-term)	This work
PRB874	*L. reuteri* with null mutant of protease harboring pLOV33	This work
PRB875	*L. reuteri* with null mutant of protease harboring pLOV33	This work
PRB876	*L. reuteri* with null mutant of protease harboring pLOV33	This work
PRB877	*L. reuteri* with null mutant of protease harboring pLOV33	This work
PRB878	*L. reuteri* with null mutant of protease harboring pLOV33	This work
PRB879	*L. reuteri* with null mutant of protease harboring pLOV33	This work
PRB880	*L. reuteri* with null mutant of protease harboring pLOV33	This work
**Plasmid**	**Description**	**Source**
pLOV67	pLS103 with double N terminal proline mutations (P2G, P17G)	This work
pLOV68	pLS103 with single N terminal proline mutation (P2G)	This work
pLOV70	pLOV33 with pro-peptide added to N-term	This work

Supplementary Table 2. **Oligonucleotides and synthetic DNA fragments used in this study**

Oligonucleotide name, or DNA block	Sequence (5'-3')	Target
**For PCR**		
**oLC_201**	agcagcccatggtatcgaagaataatcgaaaggaac	Fwd, 5'-end (signal peptide LAR_0089) from gLOV_001
**oLC_689**	ggtaccgaattcctatcagatacatgcgtt	Rev, 3' -end (hIL-22) from gLOV_001
**pLS_102_2**	ggagattttagccatggagaagaaaattatttcagctattttaat	Fwd, 5'-end gLS103, Lp_3050 signal peptide
**pLS103_5**	gtgaactaattggagcagcagctgaagcttgag	Rev, 3' -end gLS103, Lp_3050 signal peptide
**pLS_103_3**	tcaagcttcagctgctgctccaattagttcacattgt	Fwd, 5'-end, hIL-22 in pLOV33
**pLS_103_6**	cgaacccggggtaccgaattcctatcagatacatgcgt	Rev, 3'-end, hIL-22 in pLOV33
**pLS_103_1**	attaaatttctccatggctaaaatctccttgtaatagtattttatagaataca	Rev, 3' pSIP411 backbone
**pLS_103_4**	gtatctgataggaattcggtaccccgggttcg	Fwd, 5' pSIP411 backbone
**pLS_107_1**	cgcatgtatctgataatgggtactgcaggcat	Fwd, 5' pNZ411 backbone
**pLS_107_2**	taaggaggcactcaccatggagaagaaaattatttcagct	Fwd, 5'-end gLS103, usp_45 signal peptide
**pLS_107_4**	taattttcttctccatggtgagtgcctccttataa	Rev, 3' pNZ411 backbone

**pLS_107_7**	atgcctgcagtacccattatcagatacatgcgttccg	Rev 3' -end gLS103, usp_45 signal peptide
**oLC_201**	agcagcccatggtatcgaagaataatcgaaaggaac	Fwd, 5' LAR_0089 signal peptide
**oLC_211**	tcgttgtggttcttctgtattagcagaagcattcatccc	Rev, 3' LAR_0089 signal peptide
**oLC_217**	gcttctgctaatacagtaacaggcgtcccggttaa	Fwd, 5'-end, Elafin in pLOV02
**oLC_213**	cagcagcgaattctaaagtccaaccggcgaga	Rev, 3'-end, Elafin in pLOV02
**For Recombineering**		
**oJP_577**	tcaaaccaccaggaccaagcgctgaaagacgacgcttTCTGCttaattcacctaatgggttggtttgatccatgaactgg	*rpo*B *L. reuteri*
**oLC_370**	taacactaacaacggctccttggacggacttataagcttgggAATTCAcgcttccccgtttaagtccgccttattcttattaaccttcgt	LAR_0023, *L. reuteri* F275
**oLC_691**	tggttcgccgttgcttgaacccgtgcatcgttgaaataaaCTTatcctccacctactaaaagtcctattataaatatccgctt	predicted protease LAR_0170
**oLC_528**	ggaaacggtcttgctttgtcctgttgaggtattgcccccaaaAGCTTagtaggcgattcccataatacacaagaacatcacggcatagaa	predicted protease LAR_0257
**oLC_540**	tgccgttcccggactttcgatataggaatttaggggccagaaAGCTTagcagcctagtaaaaggacaagccctaaaataattcctattcg	LAR_0619
**oLC_531**	tttttggacccattcctattgaaaattcacgaaccagaattcGCTAGcgtttagcaaaataataatgaccatattcatgaacaaggacaa	LAR_0665
**oLC_539**	taagtccgccttattcttattaaccttcgtaccaccagacttGGGCTagccagctgggactcgtgtacttgttacttcttcatgatgttg	LAR_1141
**oLC_690**	aataattttgtaattgaagccaccggaagagccttatctacTCAAAgcttatagataatttgccccgttgaagcatcagccatgatcgct	LAR_1726
**DNA Synthetic fragment**		

**gLOV_001**	Agattttagccatggtatcgaagaataatcgaaaggaacaattccggaaacaagagccgaaaaagcaacgttttgcaattaaaaagctcactgtcggagttgcttcagtccttattggttttacattcatggggatgaatgcttctgctaatacagccccaattagttcacattgtagactcgataagtcaaacttccaacagccttacatcactaaccgcacattcatgcttgctaaagaagcaagcttggccgataataacacagacgtacgactgattggtgaaaagttatttcacggagtgagcatgtctgagaggtgttatctgatgaagcaagttcttaatttcactttggaagaggtgctctttccacagtctgatcggttccaaccttacatgcaggaagttgtgccctttctggctcggttatccaatcgtttaagtacctgccatattgagggagatgaccttcacatccaaagaaacgtacagaagctcaaagacactgtcaagaaactgggcgaatccggggagatcaaggctattggcgagttagacttacttttttatgtcattacggaacgcatgtatctgataggaattcggtacccc	Template for SP:hIL-22
**gLS103**	attacaaggagattttagccatggagaaatttaattttaaaactatgttattattagttttagcttcatgtgtttttggtgttgttgttaatgttactacttcattaggtccacaaactgctattactgctcaagcttcagctgctgctccaattagttcacattg	Lp_3050 signal peptide from *L. plantarum*, codon optimized for *L. reuteri*
**gLS102**	attacaaggagattttagccatggagaagaaaattatttcagctattttaatgtcaactgttattttatcagctgcagctccattatcaggtgtttatgctgctgcagctccaattagttcacattg	*usp45 L. lactis B1:D34*
